# Gram-Scale Synthesis and Optical Properties of Self-Trapped-Exciton-Emitting Two-Dimensional Tin Halide Perovskites

**DOI:** 10.3390/nano15110818

**Published:** 2025-05-28

**Authors:** Yifeng Xing, Jialin Yin, Yifei Qiao, Jie Zhao, Haiyang He, Danyang Zhao, Wanlu Zhang, Shiliang Mei, Ruiqian Guo

**Affiliations:** Institute of Future Lighting, Academy for Engineering and Technology, College of Intelligent Robotics and Advanced Manufacturing, Fudan University, Shanghai 200433, China; 22210860083@m.fudan.edu.cn (Y.X.); 23210720024@m.fudan.edu.cn (J.Y.); 24210722208@m.fudan.edu.cn (Y.Q.); 23210720135@m.fudan.edu.cn (J.Z.); 22110720032@m.fudan.edu.cn (H.H.); 23210720026@m.fudan.edu.cn (D.Z.); fdwlzhang@fudan.edu.cn (W.Z.)

**Keywords:** two-dimensional, tin halide perovskite, self-trapped exciton, luminescent properties, white light-emitting diodes

## Abstract

Lead halide perovskites (LHPs) have superior luminescent properties, but their toxicity hinders their commercialization, arousing interests in tin halide perovskites as environmentally friendly substitutes for LHPs. Herein, we synthesized a series of two-dimensional tin halide perovskite ODASnBr_4-x_I_x_ (ODA denotes 1,8-octanediammonium, X = 0, 1, 2, 3, 4) microcrystals via an aqueous-phase method. The differences between ODASnI_4_ and ODASnBr_4_ in luminescent properties and morphological characteristics were systematically discussed for the first time and attributed to light-driven ligand-to-metal charge transfer. The prepared ODASnBr_4_ has a PL peak at 567 nm and a PL QY of 99%, and the white light-emitting diodes fabricated with ODASnBr_4_ and commercial blue phosphors realized a luminous efficacy of up to 96.27 lm/W, which demonstrated the remarkable potential of ODASnBr_4_ microcrystals for high-efficiency white light-emitting diode applications.

## 1. Introduction

Lead halide perovskites (LHPs) have been widely studied in this century due to their significant luminescent properties, including high color purity, tunable bandgap, and high photoluminescence quantum yield (PL QY), demonstrating promising prospects in fields such as electroluminescence [1,2,3,4,5,6], solar cells [7,8], photodetectors [9], and bioimaging [10]. However, the severe biotoxicity of lead impedes the commercialization of LHPs. Tin is an element of Group IVA and is analogous to lead. As the degradation products of tin halide perovskites (THPs) are mostly low-toxic, such as tin oxides and tin halides, extensive studies have been inspired on THPs as eco-friendly alternatives to LHPs in recent years.

In contrast to conventional band-edge-to-band-edge emission, some two-dimensional (2D) THPs exhibit a distinct luminescent behavior known as self-trapped exciton (STE) emission [11]. Such luminescent behavior originates from localized excitons induced by lattice distortion, which typically requires the coexistence of low lattice deformation energy and strong exciton–phonon coupling [12]. Consequently, STE emission is mainly observed in zero-dimensional perovskites, whereas in 2D THPs, such emission has only been reported in perovskites incorporating specific A-site cations. STE emission usually exhibits a PL spectrum with wide full width at half maximum (FWHM) and high PL QY. Wang et al. first reported the synthesis of OA_2_SnX_4_ (OA denotes octylammonium, X = Br, I) in acidic aqueous solution [13]. The prepared microcrystal has a PL QY as high as 95% and a broadband spectrum covering yellow and red spectrum regions. Li et al. presented an air ambient method for the synthesis of 2D THP (RNH_3_)_2+x_SnI_4+x_ [14]. The PL QY of HA_2_SnI_4_ (HA denotes hexylammonium) was increased from less than 1% to 99% (HA_2+x_SnI_4+x_), and the white light-emitting diodes (WLEDs) fabricated with HA_2+x_SnI_4+x_ had Commission Internationale de l’Eclairage (CIE) coordinates at (0.447, 0.383), a correlated color temperature (CCT) of 2654 K, and a high color rendering index (CRI) of 92. Chen et al. discussed the effect of exciton localization on enhancing STE emission in 2D THPs [15]. They proposed a facile vacancy tuning strategy by changing the feeding ratio of amine and tin precursors. The PL QY of OA_2_SnI_4_ increased from approximately 1% to 63.6%, which was one of the best records in tin iodide perovskites. Recent reports on 2D THPs with STE emission are summarized in Table 1. As one of the most studied A-site cations, 1,8-octanediammonium (ODA^2+^) can naturally induce STE emission in 2D THPs. In addition, some previous reports demonstrated the outstanding optical properties of ODASnBr_4_. However, as an emerging material, ODASnBr_4_ has not been widely explored for its potential in WLED applications.

Furthermore, experiments have demonstrated that halogen ions can also impact the formation of STE emission. Chen et al. synthesized halogen-ion-tunable PEA_2_SnX_4_ (PEA denotes phenylethylammonium, X = Br, I), whose FWHM can be widened from 36 nm (PEA_2_SnI_4_) to 80 nm (PEA_2_SnBr_4_) [24]. Yao et al. synthesized TMPDA_2_SnX_4_ (X = Cl, Br, I; TMPDA denotes N,N,N′,N′-tetramethyl-1,4-phenylenediammonium) [25]. Interestingly, TMPDA_2_SnI_4_ and TMPDA_2_SnBr_4_ were both narrowband-emitting 2D THPs, but TMPDA_2_SnCl_4_ naturally formed a one-dimensional five-coordinated pyramid configuration and exhibited STE emission, which was attributed to the long Sn···Cl bond. Notably, studies have revealed that in metal halide perovskites, I^−^ ions exhibit significantly stronger suppression of lattice distortion compared to Br^−^ ions and Cl^−^ ions, which is in line with their bigger ionic radius [26]. Therefore, STE emission in tin–iodide perovskites is comparatively rare. In addition, the larger ionic radius of I^−^ ions compared to Br^−^ ions makes it easier to be oxidized [27,28], resulting in the formation of tin vacancies and a low PL QY in tin iodide perovskites.

Herein, an aqueous-phase method was employed for the synthesis of STE-emitting DJ phase ODASnBr_4−x_I_x_ microcrystals. Effects of different halogen ions on the optical properties of the perovskite are confirmed via X-ray photoelectron spectroscopy (XPS) and Fourier-transform infrared spectroscopy (FTIR) characterization, and it turns out that bi-oxidation of Sn^2+^ and I^−^ is the cause of the low PL QY in ODASnI_4_ perovskites. The prepared ODASnBr_4_ microcrystal exhibits a broadband emission peak at 567 nm (FWHM~114 nm) with a near-unity PL QY and remarkable stability. Finally, a series of WLEDs with tunable correlated color temperature (CCT) are prepared by encapsulating ODASnBr_4_ and commercially available blue phosphor on a 365 nm ultraviolet (UV) chip under different blending ratios, with a luminous efficacy (LE) up to 96.27 lm/W.

## 2. Materials and Methods

### 2.1. Materials

Stannous oxide (SnO, 99.9%), hypophosphorous acid (H_3_PO_2_, 50%), hydroiodic acid (HI, 55–58%), hydrobromic acid (HBr, 48%), octylenediamine (C_8_H_20_N_2_, 98%), tin(II) bromide (SnBr_2_, 99%), stannous oxalate (SnC_2_O_4_, 98%), and stannous octoate (C_16_H_30_O_4_Sn, 95%) are purchased from Aladdin (Waukesha, WI, USA). Ethyl acetate (C_4_H_8_O_2_, AR) is purchased from Sinopharm (Beijing, China). All chemicals are used without purification unless otherwise noted.

### 2.2. Synthesis of ODASnI_4_ Perovskite Microcrystals

For the synthesis of ODASnI_4_ microcrystals, SnO (0.1347 g), ODA (0.1731 g), HI solution (4 mL), and H_3_PO_2_ solution (13.5 mL) are added into a 100 mL flat-bottomed glass beaker and stirred at 600 rpm in order that the precursors can dissolve completely. Subsequently, the solution is heated from room temperature to 100 °C within 5 min and maintained at this temperature for 30 min. After that, the solution is allowed to cool down naturally and is ultrasonically treated for 10 min to facilitate the crystallization of ODASnI_4_ microcrystals.

### 2.3. Synthesis of ODASnBr_4−x_I_x_ Perovskite Microcrystals

The synthesis of ODASnBr_4−x_I_x_ microcrystals is similar to that of ODASnI_4_, except that HI solution in the precursor is replaced with HBr solution. For x = 1, 2, 3, 4, the HBr solution and HI solution used are (4 − x) mL and x mL, respectively.

### 2.4. Preparation of ODASnI_4_-BMA WLED

For the preparation of ODASnI_4_-BMA WLED, the phosphor, Part A glue (Epoxy resin) and Part B glue (Hardener) are uniformly mixed at a mass ratio of 20:1:4 and then encapsulated onto a 365 nm UV chip and cured under UV light for one hour. The phosphor is composed of ODASnBr_4_ microcrystals and commercially available BaMg_2_Al_16_O_27_:Eu^2+^ (BMA) phosphor blended at different mass ratios.

### 2.5. Characterization

Photoluminescence (PL) spectra and photoluminescence excitation (PLE) spectra of the prepared 2D THP microcrystals are recorded using an F97XP fluorescence spectrophotometer. Time-resolved photoluminescence (TRPL) decay curves of the perovskite powders are recorded using an FLS920 spectrophotometer. Chemical valence state and concentration of each element are determined by an Escalab Xi+, Thermo Fisher (Waltham, MA, USA), with a minimum resolution of 0.1 eV. The binding energy calibration is based on C 1s at 284.8 eV. The X-ray diffraction (XRD) patterns of the 2D THPs were recorded using a D8VENTURE MetalJet X-ray diffractometer equipped with Cu K α radiation (k = 1.54178 Å), with the voltage and current set to 40 kV and 40 mA, respectively. The morphology of the powder 2D THPs and element mapping images are obtained using an S4800 scanning electron microscope (SEM) operating at 15.0 kV. The diffuse reflectance spectra of perovskite microcrystals are recorded using a PE Lambda 750. The FTIR spectroscopy is recorded by using Nicolet Summit X (Thermo Fisher, Waltham, MA, USA). Photographs are taken by using the Vivo Neo 10.

## 3. Results and Discussion

The large size of iodide ions inhibits the deformation of the inorganic layer as well as the appearance of STEs. Therefore, there are few relevant reports of 2D tin–iodide perovskites exhibiting STE emission. Considering that the soft straight-chain structure of long-chain aliphatic organic molecules promotes the localization of excitons, we choose ODA^2+^, a kind of bivalent amine, as the A-site cation of the target DJ-phase 2D THP. The amine group of ODASnI_4_ is connected with the halogen ions by hydrogen bonding, which results in a shorter interlayer spacing as well as better stability of the perovskite compared to RP-phase 2D perovskites such as OA_2_SnI_4_ [15].

**Figure 1:**
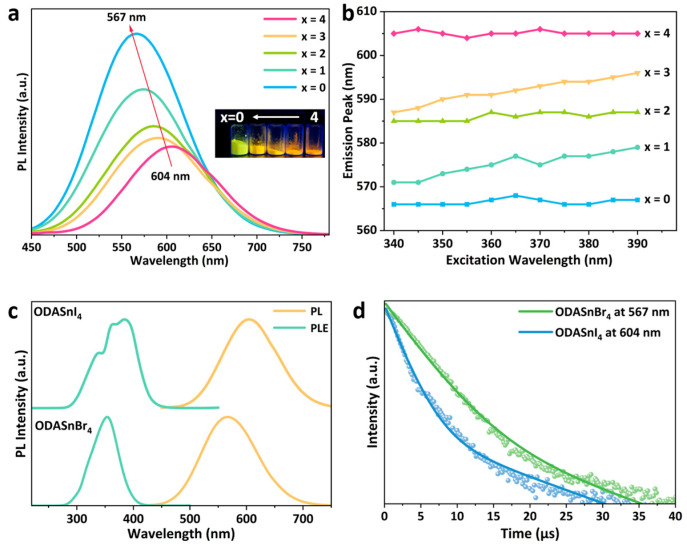
(**a**) PL spectra of ODASnBr_4−x_I_x_; the inset is the photograph of ODASnBr_4-x_I_x_ under 395 nm UV light. (**b**) PL peaks of ODASnBr_4−x_I_x_ under different excitations. (**c**) PL and PLE spectra of ODASnI_4_ and ODASnBr_4_ microcrystals. (**d**) TRPL decay curves of ODASnI_4_ and ODASnBr_4_.

Using an aqueous-phase method, the microcrystalline 2D THP ODASnBr_4-x_I_x_ (x denotes the amount of hydrohalic acid in the precursor, x = 0, 1, 2, 3, 4) were successfully synthesized by gradually adjusting the type of halogen in the precursor solution. As the HI in the precursor was gradually replaced by HBr, the PL peak of ODASnBr_4−x_I_x_ became progressively stronger, with a systematic blueshift from 604 nm (x = 4) to 567 nm (x = 0) (Figure 1a). It is worth noting that ODASnBr_4_ can crystallize naturally in an ambient environment, whereas iodine-containing ODASnBr_4−x_I_x_ needs to precipitate with the assistance of ultrasonic treatment, which implies that Br^−^ has a stronger coordination ability for Sn^2+^ than I^−^. According to previous reports, the bromine–iodine substitution in THPs has a significant effect on the crystallization kinetics of perovskites [29]. The metal sites (Sn^2+^) are more readily coordinating with Br^−^, which usually makes the actual bromine–iodine ratio in perovskites higher than the feed ratio.

In addition, to verify that the synthesized perovskite is alloyed ODASnBr_4-x_I_x_ rather than a simple mixture of ODASnBr_4_ and ODASnI_4_, the PL spectra of ODASnBr_4-x_I_x_ under different excitations were further tested (Figure 1b and Figure S1). It can be seen from the variable excitation PL spectra that the emission peak wavelengths of ODASnBr_4_, ODASnBr_2_I_2_, and ODASnI_4_ are almost fixed, while those of ODASnBr_1_I_3_ and ODASnBr_3_I_1_ exhibit a redshift of up to about 10 nm with the change in the excitation from 340 nm to 390 nm. The excitation-independent PL emission exhibited by ODASnBr_2_I_2_ demonstrates that the alloying of Br^−^ and I^−^ forms a more homogeneous band structure. ODASnBr_1_I_3_ and ODASnBr_3_I_1_ exhibit a slight excitation-dependent phenomenon, implying that there is a localized aggregation of Br^−^ and I^−^ in the lattice, which results in different PL contributions under different excitations. Considering the good symmetry of the PL spectra of ODASnBr_4−x_I_x_ and no occurrence of obvious dual emission phenomenon, it can be confirmed that the synthesized perovskites are halogen-alloyed ODASnBr_4−x_I_x_ with different bromine/iodine ratios instead of a simple mixture of ODASnBr_4_ and ODASnI_4_ microcrystals.

It can be observed from the PL spectra that there is a significant difference between the PL peaks of ODASnI_4_ and ODASnBr_4_ (Figure 1c), which may be caused by the decrease in the radius of halogen ions, the contraction of the inorganic layer, and the increase in the bandgap during the substitution of the halogen from iodine to bromine [24,25]. ODASnBr_4_ and ODASnI_4_ microcrystals are both large in size and cannot be well dispersed in solution for absorption characterization. As a result, their UV–visible diffuse reflectance spectra were collected and converted into absorption spectra (Figure S2) by using the Kubelka–Munk (K-M) formula, which is shown in Equation (1).

F(R) = K/S = (1 − R)^2^/(2R) (1)

where K is the absorption coefficient, S is the reflection coefficient, and R is the relative reflectivity of the approximately infinitely thick sample.

The absorption spectra can be used for obtaining the approximate bandgaps of the two mentioned perovskites by Tauc plot, which is shown in Equation (2).

(αhν)^(1/n) = B(hν − E_g_) (2)

where α is the absorption coefficient; h is Planck’s constant (h ≈ 4.13567 × 10^−15^ eV s); ν is the incident photon frequency (in ν = c/λ, c is the speed of light, c ≈ 3 × 10^8^ m/s, and λ is the wavelength of the incident light); n is a constant associated with the type of semiconductor material (n is 0.5 for materials of direct bandgap and 2 for that of indirect bandgap); B is a constant of proportionality; and E_g_ is the bandgap of the characterized semiconductor material.

When the absorption spectrum is converted by the Tauc plot, the section near the beginning can be fitted as a straight line, and its transverse intercept with the *x*-axis is the bandgap of the target material. The bandgaps of ODASnBr_4_ and ODASnI_4_ are 2.91 eV and 2.79 eV, respectively (Figure S3), which is in accordance with Vegard’s law [30]. However, the observed variation trend in bandgap remains consistent with experimental observations, which explains the blueshift of PL peaks from ODASnI_4_ to ODASnBr_4_.

The PL QY of ODASnI_4_ microcrystals reached 17% (Figure S4). As a comparison, ODASnBr_4_ microcrystals have a near-unity PL QY (99%), which exceeds the vast majority of 2D THPs mentioned in the Section 1 (Figure S5). It is worth noting that the PL QYs of perovskite samples are given by the integrating sphere method, as is widely recognized for the calculation of PL QY [31,32]. The remarkable difference in PL QY of ODASnI_4_ and ODASnBr_4_ is mainly caused by light-driven ligand-to-metal charge transfer (LD-LMCT), which manifests as the mutual oxidation of Sn^2+^ and I^−^ in tin–iodide perovskites, exacerbating the oxidation issue and negatively affecting the luminescent performance of ODASnI_4_ [33]. Under ambient conditions, the oxidized Sn^4+^ undergoes electron exchange with I^−^, resulting in the reduction from Sn^4+^ to Sn^2+^ and oxidation from I^−^ to I_2_. Nevertheless, the mentioned redox reaction proceeds in the reverse direction under light irradiation. Part of the Sn^2+^ cations re-oxidize to Sn^4+^, which subsequently reacts with I^−^, atmospheric water, and oxygen to form HI and SnO_2_. The generated HI further undergoes oxidation to produce I_2_ again. Such chain-like oxidation processes lead to rapid degradation of the pristine lattice through the formation of vacancy defects, thereby significantly compromising the luminescent properties of the tin–iodide perovskites.

The TRPL decay curves of ODASnI_4_ and ODASnBr_4_ are shown in Figure 1d, with the fitted lifetimes listed in Table 2. In the case of STE emission, the short exciton lifetime τ_1_ usually originates from the donor–acceptor pair recombination after the capture of carriers by surface defects, while the long exciton lifetime τ_2_ is based on the emission of STEs [34]. It is obvious that the proportion of surface defect capture in ODASnI_4_ is higher than that in ODASnBr_4_, and τ_1_ of ODASnI_4_ is also smaller than that of ODASnBr_4_, according to the calculated exciton lifetime. It is indicated that the oxidation phenomenon of ODASnI_4_ is more severe, with more tin vacancies on the surface, which enhances the non-radiative composites.

To further verify the existence of LD-LMCT, high-resolution (HR) XPS spectra of ODASnI_4_ and ODASnBr_4_ for specific elements were characterized, in which the cyan solid lines represent the fitted spectra, and the black dashed lines represent the actual measured spectra. Fitting parameters of XPS spectra have been listed in Table S1.

**Figure 2:**
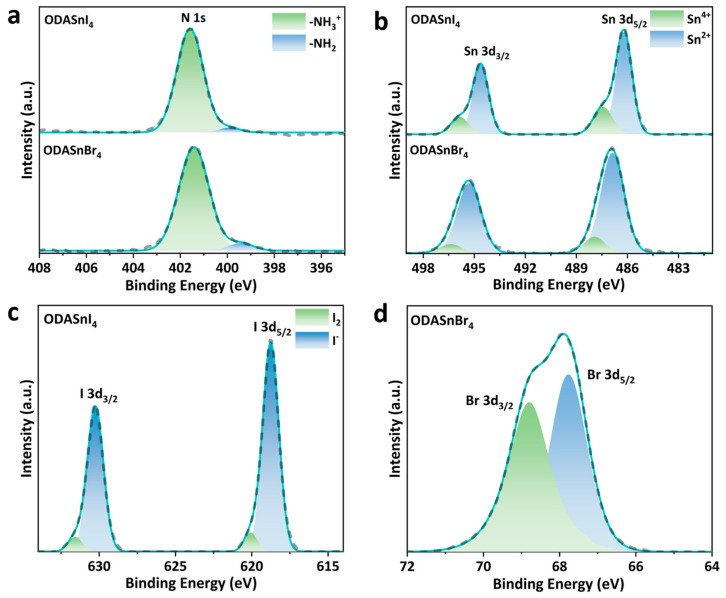
HR XPS spectra of (**a**) N 1s and (**b**) Sn 3d in ODASnI_4_ and ODASnBr_4_. HR XPS spectra of (**c**) I 3d in ODASnI_4_ and (**d**) Br 3d in ODASnBr_4_.

The overall binding energy of N 1s in ODASnI_4_ is slightly higher than that in ODASnBr_4_, which may be caused by the alternation in coordination environments resulting from the use of different halogen ions I^−^ and Br^−^ (Figure 2a) [35]. The high-energy peak of the N 1s located near 401 eV and the low-energy peak near 400 eV correspond to -NH_2_^+^ and -NH_3_, respectively, suggesting a small amount of detached ODA [36]. This may originate from the excess precursor or the formation of halogen ion vacancies from the oxidation of I^−^ and hydrogen bonding breakage leading to ODA^2+^ detachment.

The HR XPS spectra of Sn 3d in ODASnI_4_ and ODASnBr_4_ exhibit two asymmetric peaks corresponding to two spin-orbit splitting sub-energy levels, Sn 3d_3/2_ and Sn 3d_5/2_ (Figure 2b). The two low-energy peaks of Sn 3d in ODASnI_4_ located at 486.19 eV and 494.63 eV correspond to Sn^2+^, while the high-energy peaks at 487.42 eV and 495.87 eV imply the existence of Sn^4+^ [37]. As a comparison, the XPS spectrum of ODASnBr_4_ indicates a similar coexistence of Sn^2+^ and Sn^4+^, but the proportion of Sn^4+^ is lower than that in the XPS spectrum of ODASnI_4_, which substantiates that LD-LMCT results in a more pronounced oxidation problem in tin–iodide perovskites compared to their bromide counterparts. As mentioned before, oxidation of Sn^2+^ results in the formation of tin vacancies, which will become the non-radiative combination centers and degrade the PL QY of THPs.

The I 3d HR XPS spectrum of ODASnI_4_ exhibits very distinct multiple peaks in both I 3d_3/2_ and I 3d_5/2_ energy levels (Figure 2c). The low-energy peaks at 618.76 eV and 630.25 eV represent I^−^ in the perovskite layer, whereas the peaks at 620.06 eV and 631.57 eV imply the transition from I^−^ to I_2_ in ODASnI_4_, which further corroborates the oxidation caused by LD-LMCT. The Br 3d HR XPS spectrum of ODASnBr_4_ shows two overlapping binding energy peaks located at 67.77 eV and 68.80 eV, corresponding to Br 3d_3/2_ and Br 3d_5/2_, respectively (Figure 2d). The splitting energy of 1.03 eV is very close to the theoretical value of 1 eV, indicating the absence of Br^−^ oxidation [38,39]. Percentages of the major elements calculated based on XPS spectra have been listed in Table S2.

It is worth mentioning that the oxidation of the internal part of ODASnI_4_ and ODASnBr_4_ microcrystals is much lower than that of the surface, according to previous reports [40], because the organic A-site cations are capable of preventing the penetration of water and oxygen. Compared to RP-phase 2D THPs (e.g., PEA_2_SnI_4_), DJ-phase 2D THPs have fewer oxidation-induced defects, which can suppress non-radiative recombination and result in higher PL QY [23].

**Figure 3:**
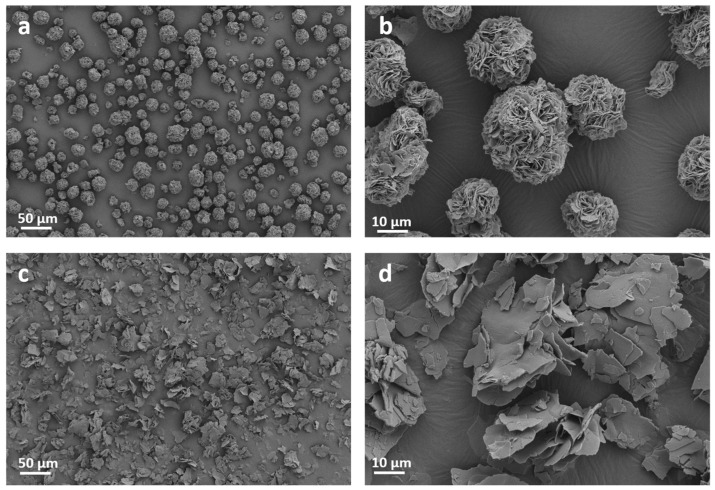
SEM images of (**a**,**b**) ODASnI_4_ microcrystals and (**c**,**d**) ODASnBr_4_ microcrystals.

Morphology characterizations on ODASnI_4_ and ODASnBr_4_ were also employed to further explore the reasons for the differences in the optical properties of these two perovskites. The interlayer spacings of ODASnI_4_ and ODASnBr_4_ calculated from XRD patterns are 1.37 nm and 1.39 nm, respectively (Figure S6). SEM images show that ODASnI_4_ and ODASnBr_4_ are both microcrystals with layered structures, which is a typical feature of 2D THPs. However, ODASnI_4_ microcrystals agglomerate into spheres (Figure 3a,b). On the contrary, the layered structure is distinct in ODASnBr_4_ samples (Figure 3c,d). The weak coordination ability of I^−^ ions reduces the passivation efficiency of the active sites on the surface of the inorganic layer, as well as causing the (SnI_6_)^4−^ octahedra to form thermodynamically more stable spherical agglomerates by adsorbing to each other via van der Waals forces.

Figures S7 and S8 show the SEM images of ODASnI_4_ and ODASnBr_4_ microcrystals and the corresponding elemental mapping images. The uniform distribution of Sn, I, and N elements confirms the generation of pure ODASnBr_4_ and ODASnI_4_ microcrystals, but the distribution of N elements at the edges of the ODASnI_4_ spheres is less. This suggests that there may be ODA detached at the edge of the inorganic layer, which prompts the (SnI_6_)^4−^ octahedra to agglomerate into spheres after mutual adsorption by van der Waals forces or halogen bonds. According to previous reports, the agglomeration of 2D perovskites can cause electronic defects and decreased quality of the perovskite crystals, as well as destroy the perpendicular quantum well orientation [41,42,43]. Furthermore, the agglomeration can also cause the optical phonon and defect scattering by reducing the screening of Coulomb potential, which leads to stronger non-radiative recombination [44].

FTIR spectra were employed to reveal the interaction of chemical bonds within ODASnI_4_ and ODASnBr_4_. There are no prominent broadband peaks from 3200 cm^−1^ to 3600 cm^−1^, which indicates the absence of significant O-H vibrations, proving that the solvent used in the reaction process is almost completely removed after purification (Figure S9). According to previous reports, the N-H stretching vibration based on the -NH_2_ group in free ODA molecules should be located at 3330 cm^−1^, whereas the N-H vibrations of both ODASnBr_4_ and ODASnI_4_ synthesized in this chapter are located at about 3090 cm^−1^, and the shift of vibrational frequency toward lower wavenumbers is attributed to the formation of hydrogen bonds between protonated ODA^2+^ cations and the inorganic layers [22,45]. Multiple peaks were observed in the range of 2800 cm^−1^ to 3000 cm^−1^ in ODASnBr_4_, and the peaks located at 2962 cm^−1^, 2928 cm^−1^ and 2854 cm^−1^ correspond to the C-H vibration of -CH_3_ in ODA, C-H symmetry vibration, and asymmetry vibration in -CH_2_-, respectively, which proves the integrity of ODA^2+^ carbon chains. Three sharper peaks can be observed in the range of 1300 cm^−1^ to 1700 cm^−1^, which corresponds to the bending vibration of -CH_3_ and correlates with the conformational ordering of the ODA, suggesting a certain degree of van der Waals’ force interaction within the organic layers, which is also in line with previous analysis in N 1s XPS spectra. According to the above analysis of N-H vibration and C-H vibration, it can be judged that ODA^2+^ cations are successfully embedded in the inorganic layer and connected to the inorganic layer via hydrogen bonding. In addition, it can be observed that the N-H peak of the ODASnI_4_ sample is more inclined to lower wavelengths compared to that of the ODASnBr_4_ sample, which is due to the stronger polarization ability of I^−^ and the formation of stronger hydrogen bonding with the organic layer. The shift of the C-H vibration can be attributed to the effect of the larger ionic radius of I^−^ on the organic layer.

ODASnBr_4_ has extremely remarkable luminescent performance, as described previously. The synthesized ODASnBr_4_ exhibits a broadband emission with the optimal excitation located at 354 nm, emission peak at 567 nm, and an FWHM of 114 nm. These luminescent properties make ODASnBr_4_ a promising candidate for WLED applications. Considering the need for commercialization, we optimized the synthesis conditions to maximize the yield while maintaining high PL QY. It should be noted that the yield of the synthesized ODASnI_4_ microcrystals was about 55% (0.4238 g), which may be caused by oxidation. Due to the LD-LMCT mechanism, tin–iodine perovskites are highly susceptible to oxidation and production of I_2_, which is soluble in ethyl acetate and is removed during purification, resulting in the yield reduction in the perovskite.

We investigated the effects of HI dosage, H_3_PO_2_ dosage, the type of tin precursors, and reaction temperature on the PL intensity and yield of ODASnBr_4_, respectively. The ODASnBr_4_ synthesized at different H_3_PO_2_ dosages exhibited different PL intensities, but their emission peaks remained near 567 nm (Figure S10). According to previous reports, the FWHM of STE emission in THPs depends on the length of the Sn-X bond, which is closely related to the synthesis environment [11]. The FWHM of ODASnBr_4_ remained constant, indicating that the aqueous-phase method applied in this article has excellent reproducibility. The highest PL intensity of ODASnBr_4_ was achieved at an H_3_PO_2_ dosage of 13.5 mL. Further increase in H_3_PO_2_ dosage leads to no further improvement in PL intensity, which indicates that the oxidation of Sn^2+^ has been completely inhibited. In addition, a rapid decrease in the yield of ODASnBr_4_ was observed under higher H_3_PO_2_ dosage. It is attributed to the fact that ODASnBr_4_ is very soluble in water, and the water introduced by additional H_3_PO_2_ could not be completely removed in the reaction, thus reducing the yield of perovskites.

In the aqueous-phase method for the synthesis of 2D THPs, an excess of hydrohalic acid is necessary to provide enough halogen ions for reducing halogen vacancies. No significant precipitation of perovskite was observed under the usage of 2 mL HI. When the amount of hydrobromic acid exceeded 3 mL, ODASnBr_4_ began to crystallize and precipitate, exhibiting the highest PL intensity at 4 mL and a gradual decrease thereafter. (Figure S11) The effect of excessive halogen ions on reducing vacancy defects has been widely recognized, which explains the increase in PL intensity as the amount of HBr rises from 2 mL to 4 mL [46,47]. Nevertheless, additional HBr introduced excessive oxygen and water into the solvent, which may induce the oxidation of Sn^2+^. Too many halogen ions may also cause deep traps and halide-rich defects, leading to the enhancement of nonradiative recombination and a decrease in optical properties with higher HBr dosages [48,49].

Different bivalent tin compounds were used as precursors. ODASnBr_4_ was synthesized using stannous oxalate (SnOAc_2_), stannous bromide (SnBr_2_), and stannous oxide (SnO). The highest PL intensity was achieved with SnO (Figure S12). Furthermore, heating is required for the synthesis of ODASnBr_4_ in the aqueous-phase method for dissolution of the precursors and precipitation of the perovskite by evaporating the solvent. ODASnBr_4_ was synthesized at different temperatures within 30 min. No precipitation of ODASnBr_4_ was observed at 80 °C, so the reaction time was extended to 60 min. Considering that too long a reaction time may lead to oxidation of Sn^2+^, no further attempts were made at synthesis temperatures below 80 °C. The PL intensity showed a positive correlation with the reaction temperature, reaching a maximum at 95 °C (Figure S13). At 100 °C, an abnormal decrease was observed in both PL intensity and yield. It may be caused by the co-evaporation of H_3_PO_2_, which has a low boiling point of 108 °C, with water, leading to the oxidation of Sn^2+^ during the reaction.

The yields of ODASnBr_4_ under different experimental conditions are calculated and listed in Tables S3. By optimizing the experimental conditions, it was possible to achieve a yield of approximately 90%. The experimental conditions of 4 mL HBr, 13.5 mL H_3_PO_2_, 1 mmol SnO, and 1.2 mmol ODA co-heated at 90 °C for 30 min were finally chosen for this article, as both the PL intensity and the yield were satisfactory under this condition. Meanwhile, due to the excellent reproducibility and simplicity of the process of the aqueous-phase method, a gram-scale production of ODASnBr_4_ microcrystals (1.3441 g) can be achieved by simply tripling the amount of precursor (Figure S14), and the yield under such conditions is 80.18%, which is a highly efficient and large-volume preparation method that is relatively rare in reports of 2D THPs.

ODASnBr_4_ has the advantage of broadband emission with high PL QY, which makes it a promising luminescent material for WLED applications. Therefore, stability tests were conducted to ensure that this kind of STE-emitting 2D THP can be used as a long-lasting phosphor after encapsulation. Based on the application scenarios of WLEDs, the thermal stability, photostability, and air stability of ODASnBr_4_ were tested, respectively.

In the thermal stability test, the ground ODASnBr_4_ microcrystals were sealed in flat-bottomed glass vials and heated for 4 h at 90 °C, which was selected because it approximates the operating conditions of a 3W LED chip. To ensure that the ODASnBr_4_ samples are not affected by other environmental conditions (e.g., oxidation due to exposure to air), the samples for characterization were weighed from continuously heated and unopened glass vials. The result exhibited that the PL intensity of ODASnBr_4_ decreased approximately linearly with time and remained at about 90% of the original PL intensity after 4 h of heating (Figure S15). In addition, the thermal stability test was also operated at 70 °C and 120 °C to simulate moderate and extreme conditions under the operation of LED. It is observed that the ODASnBr_4_ microcrystals exhibited a similar linear downward trend in PL intensity under both temperatures, which further verified their good thermal stability.

In the photostability test, a 16 W UV light was used for photo-aging. The PL intensity of ODASnBr_4_ exhibited a significant decrease in the first hour but gradually stabilized in the subsequent tests and remained 68.2% of the original intensity after being continuously irradiated for 72 h (Figure S16).

In addition, ODASnBr_4_ powder was placed in a room-temperature ambient environment for 72 h. The PL intensity of ODASnBr_4_ decreased slowly and linearly with time and retained 89.90% of the initial PL intensity after 72 h (Figure S17). It is worth noting that the wavelength changes in the emission peaks remained within 3 nm during previous stability tests, indicating remarkable color stability of ODASnBr_4_ (Figure S18).

**Figure 4:**
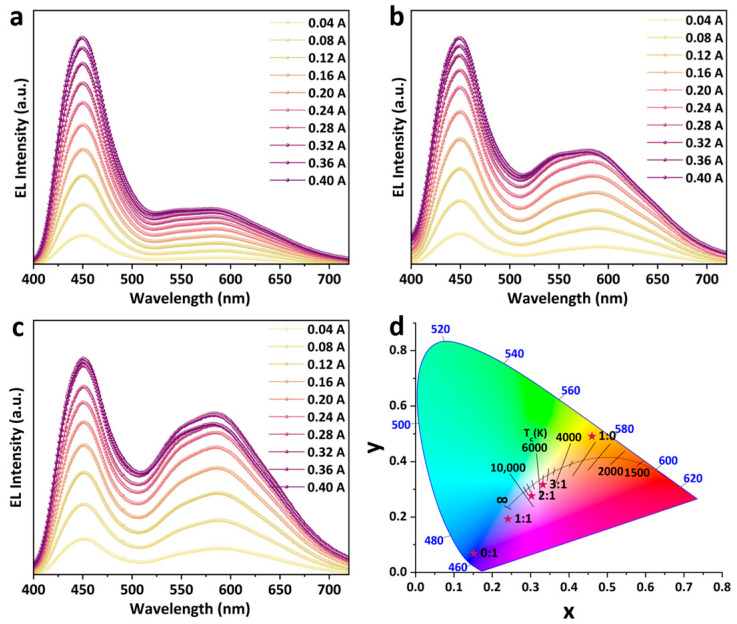
EL spectra of WLEDs prepared with ODASnBr_4_ and BMA phosphors at blending ratios of (**a**) 1:1, (**b**) 2:1, and (**c**) 3:1 under different driving currents. (**d**) CIE chromaticity coordinates of ODASnBr_4_, BMA phosphor, and the WLEDs prepared with different blending ratios. Red stars correspond to CIE coordinates of WLEDs with different blending ratios (annotated adjacent to each red star).

The synthesized ODASnBr_4_ microcrystals with broadband yellow emission were mixed with commercially available BaMg_2_Al_16_O_27_:Eu^2+^ (BMA) blue phosphor, then encapsulated on a 365 nm UV chip, with a view to obtaining a two-component WLED with excellent LE and Ra. A series of WLEDs with different EL spectra were prepared by varying the blending ratios of ODASnBr_4_ microcrystals and BMA phosphor. It can be observed that the EL intensity of both phosphors increases steadily with increasing current, and the increase in blending ratio of ODASnBr_4_/BMA from 1:1 to 3:1 can significantly enhance the EL intensity of ODASnBr_4_ (Figure 4a–c). Varying the blending ratio of ODASnBr_4_ and BMA can significantly move the color coordinates of the prepared WLEDs from (0.2406, 0.1928) (1:1) to (0.3023, 0.2761) (2:1) and (0.3321, 0.3167) (3:1) (Figure 4d).

**Figure 5:**
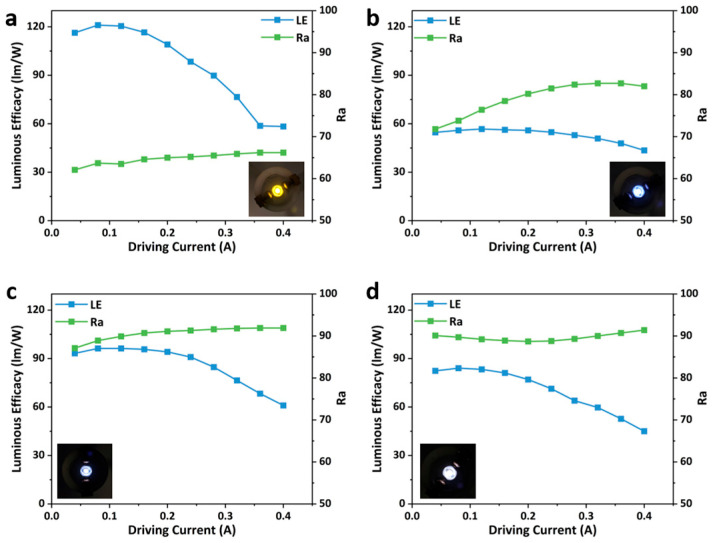
Luminous efficacy and CRI of the prepared WLEDs with blending ratios of ODASnBr_4_ and BMA phosphors of (**a**) 1:0, (**b**) 1:1, (**c**) 2:1, and (**d**) 3:1 under different driving currents. The insets are photographs of the correlated LEDs under a current of 0.1 A.

The LE and Ra of the LEDs prepared with different ODASnBr_4_/BMA blending ratios also exhibited regular changes with the driving current. Notably, the LE of ODASnBr_4_ reached up to 121.05 lm/W when encapsulated on the UV chip alone (Figure 5a), and that of WLEDs with blending ratios of 1:1, 2:1, and 3:1 exhibited a measurable reduction, which might originate from the energy transfer between ODASnBr_4_ and BMA. The EL spectra of the prepared WLEDs were more homogeneous due to the blending of blue phosphor, thus leading to a distinct increase in Ra (Figure 5b–d). WLEDs with a blending ratio of 2:1 exhibited the best LE and Ra compared to the WLEDs with different blending ratios. The WLED with a blending ratio of 2:1 has a highest Ra of 91.9, and its LE reaches up to 96.27 lm/W under a driving current of 0.12 A (Table S4).

The WLED with a blending ratio of 2:1 was placed under a driving current of 100 mA for 40 min, and the luminescent data were recorded every minute. After 40 min, the EL intensities of both BMA and ODASnBr_4_ slightly decreased. LE of the WLED showed a nearly linear decrease and remained 93.24% of the original LE after 40 min (Figure S19). The CIE coordinates of the WLED showed only a slight drift from the original (0.3043, 0.2680) to (0.3040, 0.2737) (Figure S20). The above results indicate that the WLEDs prepared via ODASnBr_4_ have excellent luminescent performance and stability, while further studies remain necessary to meet the demand of commercialization.

## 4. Conclusions

In summary, ODASnI_4_ and ODASnBr_4_ microcrystals were synthesized by a facile aqueous-phase method. The effect of LD-LMCT on these two perovskites is exposed by discussing their difference in optical properties as well as morphology, which is mainly caused by the co-oxidation of Sn^2+^ and I^−^. The optimization of synthesis conditions obtained ODASnBr4 microcrystals with high yield (approximately 90%), broadband yellow emission, near-unity PL QY, and remarkable stability. After that, the WLEDs prepared with ODASnBr_4_ microcrystals and BMA phosphors exhibited an Ra of 91.9 and a maximum LE of over 96 lm/W. This article systematically discussed the optical properties of ODASnX_4_ (X = Br, I), highlighting their potential in high-performance, environmentally friendly solid-state lighting applications.

## Figures and Tables

**Table 1:** Summary of recent reports on STE-emitting 2D THPs.

Year	Materials	Emission Peak Wavelength/nm	FWHM/nm	PL QY	Ref.
2019	(OA)_2_SnBr_4_	600	136	95%	[13]
2019	(OAm)_2_SnI_4_	620	140	88%	[16]
2019	(RNH_3_)_2_SnBr_4_	612–628	126–156	1.94–61.08%	[17]
2021	(RNH_3_)_2_SnBr_4_:Zn	580	137	85%	[18]
2021	ODASnBr_4_	570–608	126–135	88%	[19]
2021	ODASnBr_4_	572–601	/	88%	[20]
2021	ODASnBr_4_	610	/	86%	[21]
2022	ODASnBr_4_	586	180	98.22%	[22]
2022	(8N8)SnBr_4_	580	130	99.7%	[23]
2022	HA_2+x_SnI_4+x_	598	126	99%	[14]
2023	OA_2_SnI_4_	655	160	63.6%	[15]
2025	ODASnBr_4_	567	114	99%	This work
2025	ODASnI_4_	604	115	17%	This work

**Table 2:** TRPL parameters of ODASnI_4_ and ODASnBr_4_ under optimal excitation.

Samples	τ_1_/μs	Rel_1_/%	τ_2_/μs	Rel_1_/%	τ_ave_/μs
ODASnI_4_	2.51	56.73	20.09	43.27	10.12
ODASnBr_4_	4.70	48.10	23.19	51.90	14.29

## Data Availability

Data are contained within this article.

## Supplementary Materials

The following supporting information can be downloaded at: https://www.mdpi.com/article/10.3390/nano15110818/s1. Figure S1. PL spectra of ODASnBr4-xIx microcrystals under different excitations when (a) x = 4, (b) x = 3, (c) x = 2, (d) x = 1, and (e) x = 0. Figure S2. Diffuse reflectance spectra of (a) ODASnI4 and (b) ODASnBr4 represented by the K-M function. Figure S3. Tauc plot of (a) ODASnI4 and (b) ODASnBr4. Figure S4. PL spectra of ODASnI4 for the calculation of PL QY. Figure S5. PL spectra of ODASnBr4 for the calculation of PL QY. Figure S6. XRD patterns of ODASnI4 and ODASnBr4. Figure S7. (a) SEM image and element mapping images of (b) Sn, (c) I, and (d) N atoms of ODASnI4 microcrystals. Figure S8. (a) SEM image and element mapping images of (b) Sn, (c) Br, and (d) N atoms of ODASnBr4 microcrystals. Figure S9. (a) FTIR spectrum and (b), (c) enlarged spectrum of ODASnX4 (X = Br, I). Figure S10. PL spectra of ODASnBr4 synthesized with different amounts of H3PO2. Figure S11. PL spectra of ODASnBr4 synthesized with different amounts of HBr. Figure S12. PL spectra of ODASnBr4 synthesized with different tin precursors. Figure S13. PL spectra of ODASnBr4 synthesized with different reaction temperatures. Figure S14. Photographs of ODASnBr4 microcrystals prepared in large quantities in different states. From left to right are photographs of (a) precursor, (b) solution after heating, (c) perovskite after crystallization, and (d) perovskite microcrystals under 365 nm UV light. Figure S15. PL spectra of ODASnBr4 as a function of time in thermal stability test under (a) 70 °C, (b) 90 °C, and (c) 120 °C. Figure S16. PL spectra of ODASnBr4 as a function of time in the photostability test. Figure S17. PL spectra of ODASnBr4 as a function of time in the air stability test. Figure S18. Relative intensity and PL peaks of ODASnBr4 as a function of time in (a) thermal (90 °C), (b) photo, and (c) air stability tests. Figure S19. (a) EL spectra and (b) LE of the prepared WLED with a blending ratio of 2:1 before and after the stability test. Figure S20. CIE chromaticity coordinates of the WLED (a) before and (b) after stability test. Table S1. Fitting parameters in XPS spectra. Table S2. Percentages of the major elements calculated based on XPS spectra. Table S3. Yield of ODASnBr4 calculated by tin under different experimental conditions; “/” represents no production. Table S4. Performance parameters of WLED prepared with ODASnBr4 and BMA under a 2:1 blending ratio.
