# Supplementary material for: Gram-Scale Synthesis and Optical Properties of Self-Trapped-Exciton-Emitting Two-Dimensional Tin Halide Perovskites
Source: Nanomaterials (Basel). 2025 May 28;15(11):818. doi: 10.3390/nano15110818 (PMC12157897; doi:10.3390/nano15110818)
Supplement: Supplementary file 1 [file nanomaterials-15-00818-s001.zip › nanomaterials-3613688-supplementary.pdf]

nanomaterials

Article

Gram-Scale Synthesis and Optical Properties of Self-Trapped-Exciton-Emitting Two-Dimensional Tin Halide Perovskites

Yifeng Xing, Jialin Yin, Yifei Qiao, Jie Zhao, Haiyang He, Danyang Zhao, Wanlu Zhang, Shiliang Mei * and Ruiqian Guo *

Institute of Future Lighting, Academy for Engineering and Technology, College of Intelligent Robotics and Advanced Manufacturing, Fudan University, Shanghai 200433, China; 22210860083@m.fudan.edu.cn (Y.X.); 23210720024@m.fudan.edu.cn (J.Y.); 24210722208@m.fudan.edu.cn (Y.Q.); 23210720135@m.fudan.edu.cn (J.Z.); 22110720032@m.fudan.edu.cn (H.H.); 23210720026@m.fudan.edu.cn (D.Z.); fdwlzhang@fudan.edu.cn (W.Z.)
* Correspondence: meishiliang@fudan.edu.cn (S.M.), rqguo@fudan.edu.cn (R.G.)

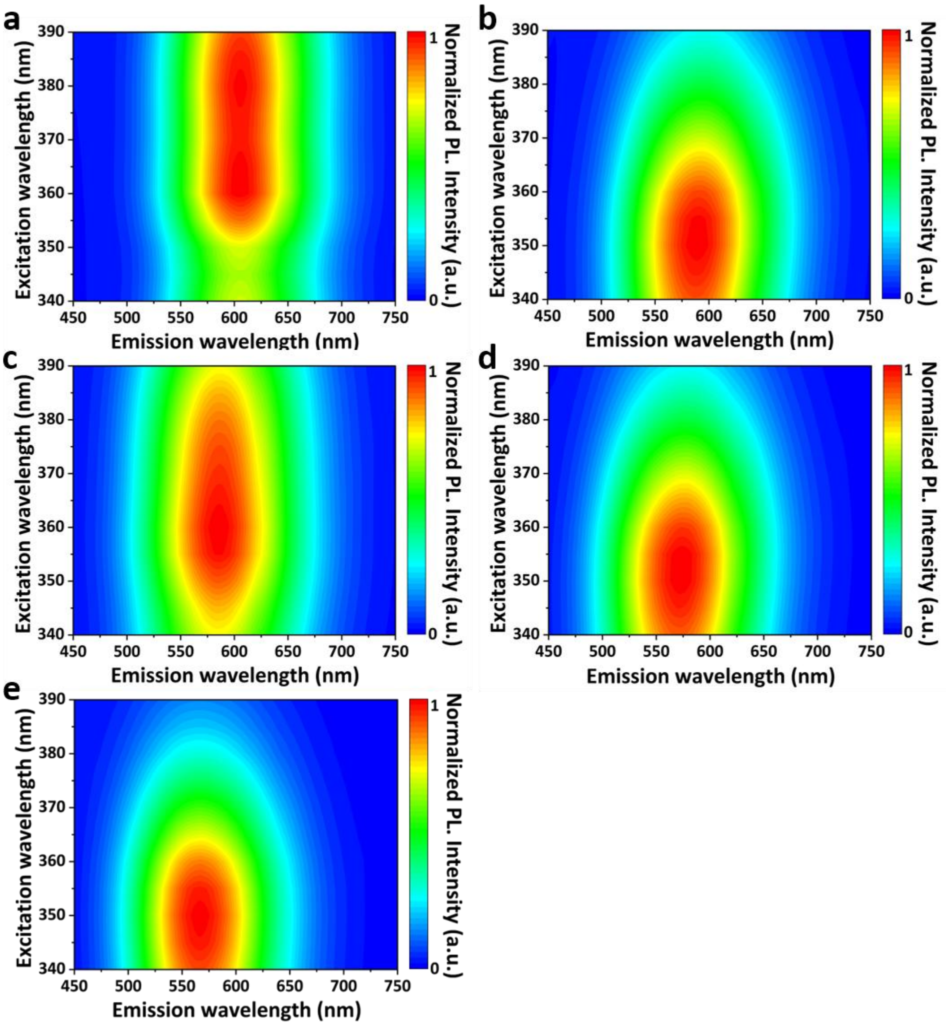

Figure S1. PL spectra of $ODASnBr_{4-x}I_x$ microcrystals under different excitations when (**a**) x = 4, (**b**) x = 3, (**c**) x = 2, (**d**) x = 1 and (**e**) x = 0

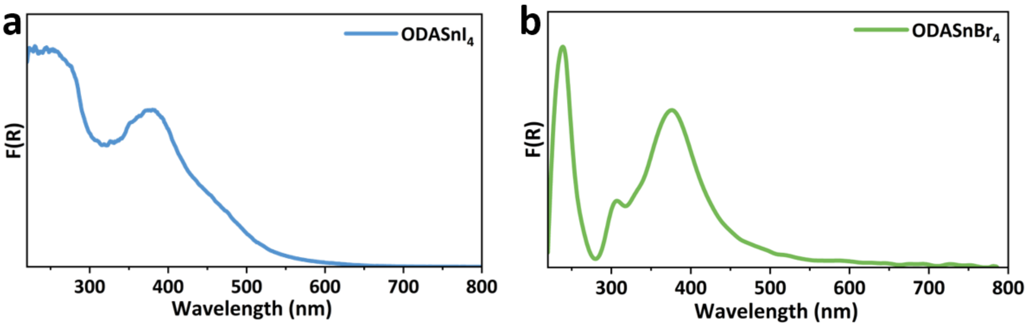

Figure S2. Diffuse reflectance spectra of (**a**) $ODASnI_4$ and (**b**) $ODASnBr_4$ represented by the K-M function.

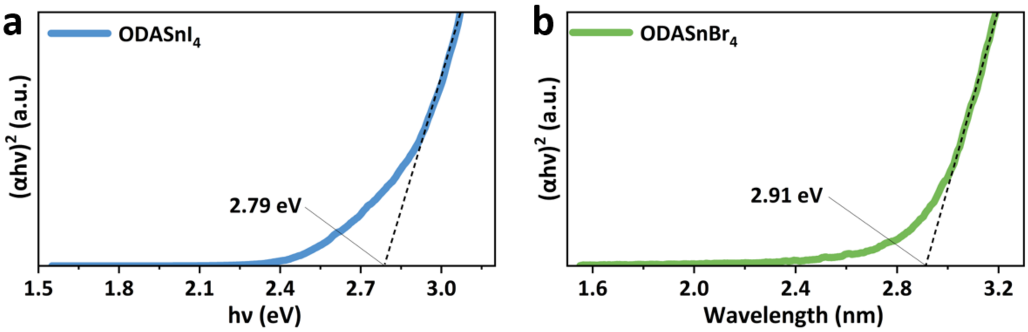

Figure S3. Tauc plot of (**a**) $ODASnI_4$ and (**b**) $ODASnBr_4$.

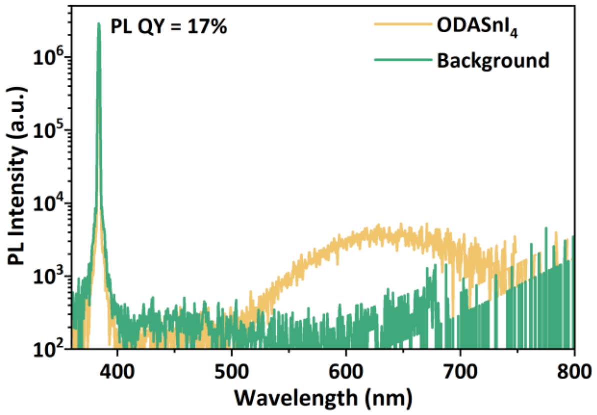

Figure S4. PL spectra of $ODASnI_4$ for the calculation of PL QY.

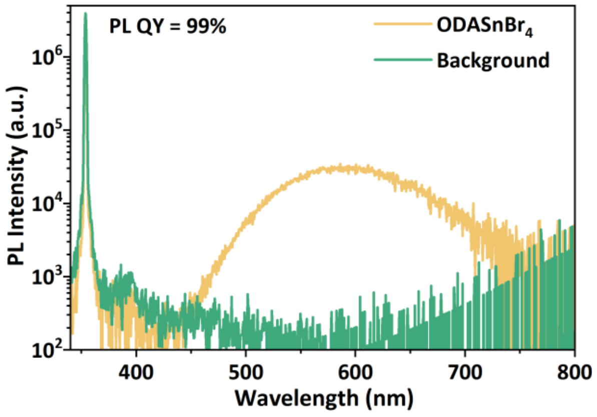

Figure S5. PL spectra of $ODASnBr_4$ for the calculation of PL QY.

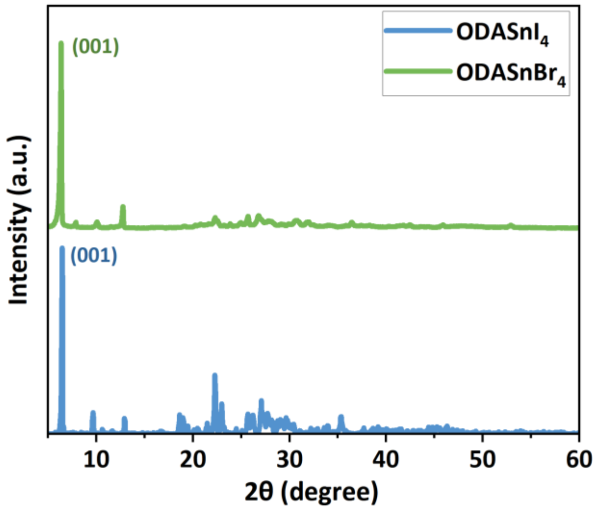

Figure S6. XRD patterns of $ODASnI_4$ and $ODASnBr_4$.

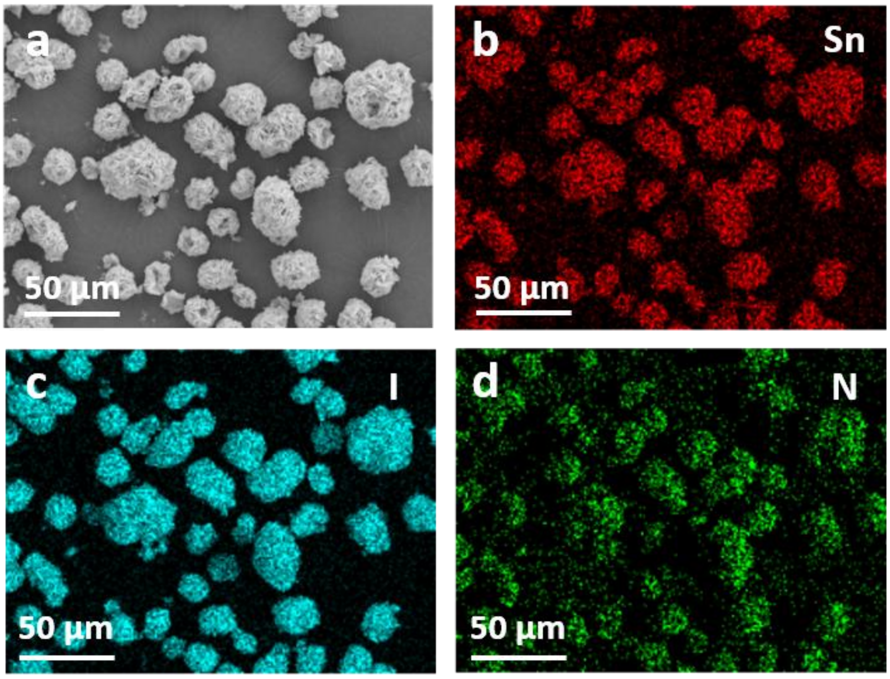

Figure S7 (**a**) SEM image and element mapping images of (**b**) Sn, (**c**) I, and (**d**) N atoms of $ODASnI_4$ microcrystals.

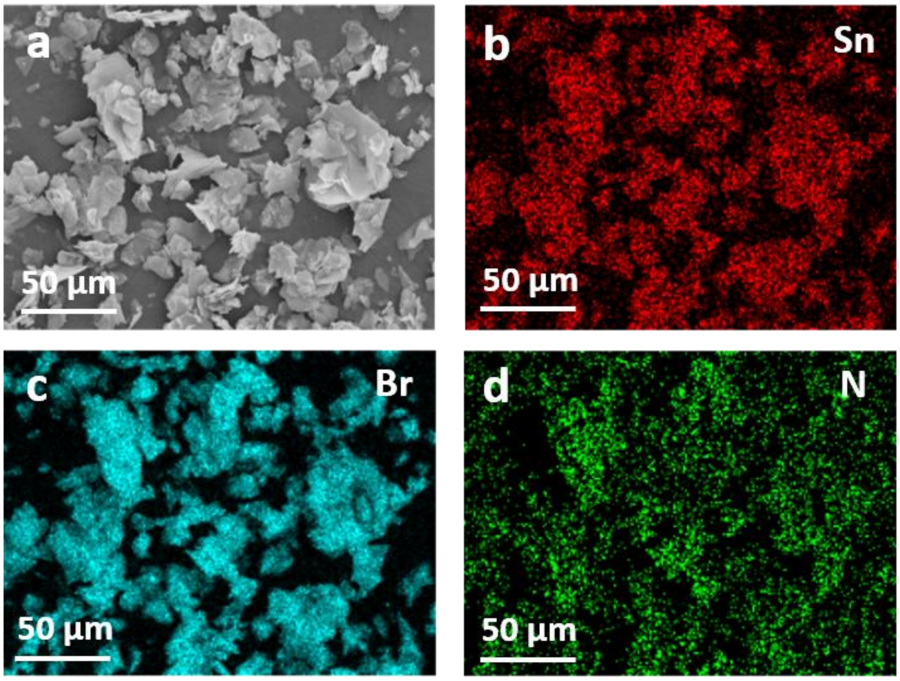

Figure S8. (**a**) SEM image and element mapping images of (**b**) Sn, (**c**) Br, and (**d**) N atoms of ODASnBr4 microcrystals.

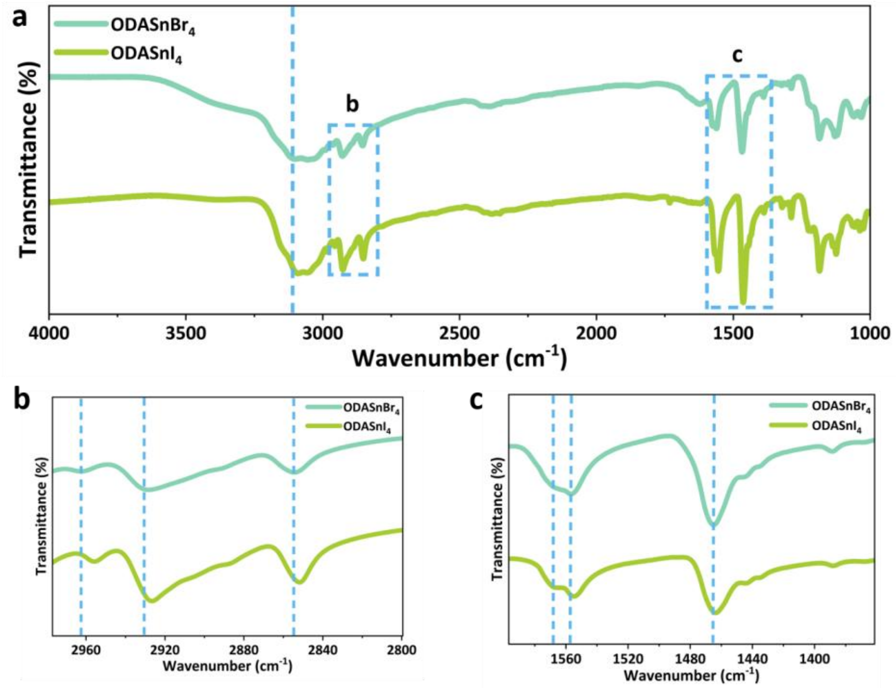

Figure S9. (**a**) FTIR spectrum and (**b**), (**c**) enlarged spectrum of ODASnX4 (X = Br, I).

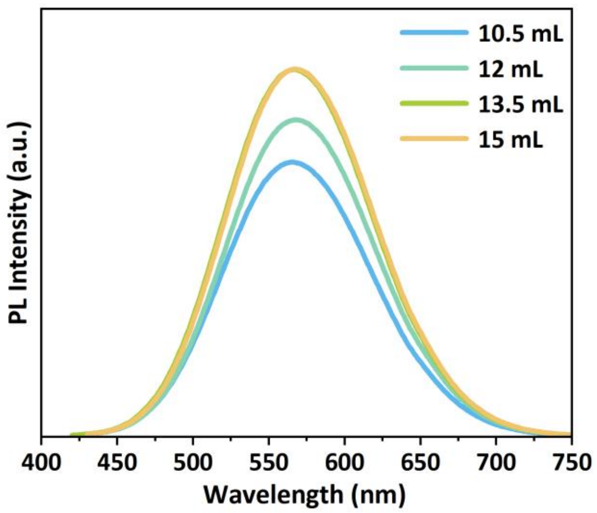

Figure S10. PL spectra of $ODASnBr_4$ synthesized with different amounts of H_3PO_2.

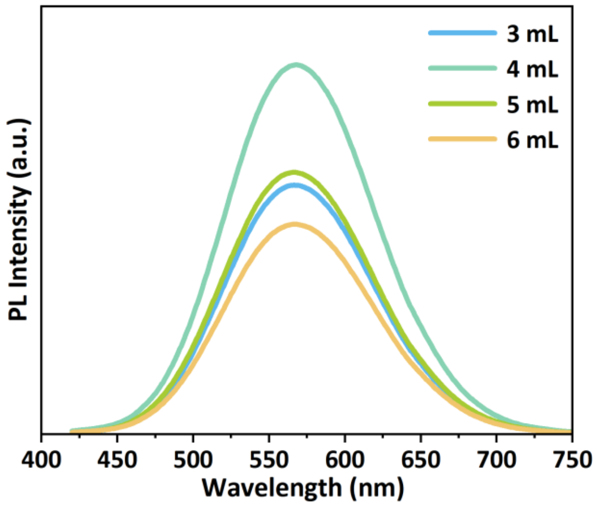

Figure S11. PL spectra of $ODASnBr_4$ synthesized with different amounts of HBr.

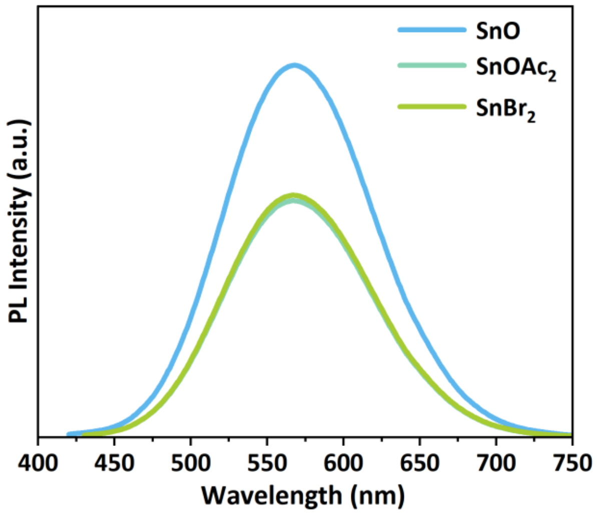

Figure S12. PL spectra of $ODASnBr_4$ synthesized with different tin precursors.

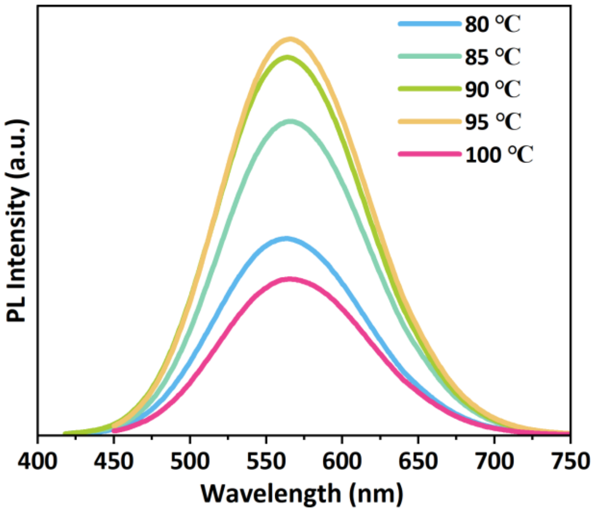

Figure S13. PL spectra of ODASnBr4 synthesized with different reaction temperatures.

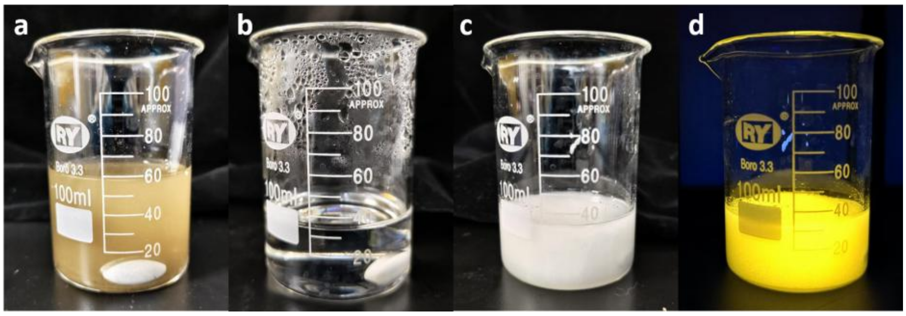

Figure S14. Photographs of ODASnBr4 microcrystals prepared in large quantities in different states. From left to right are photographs of (**a**) precursor, (**b**) solution after heating, (**c**) perovskite after crystallization and (**d**) perovskite microcrystals under 365 nm UV light.

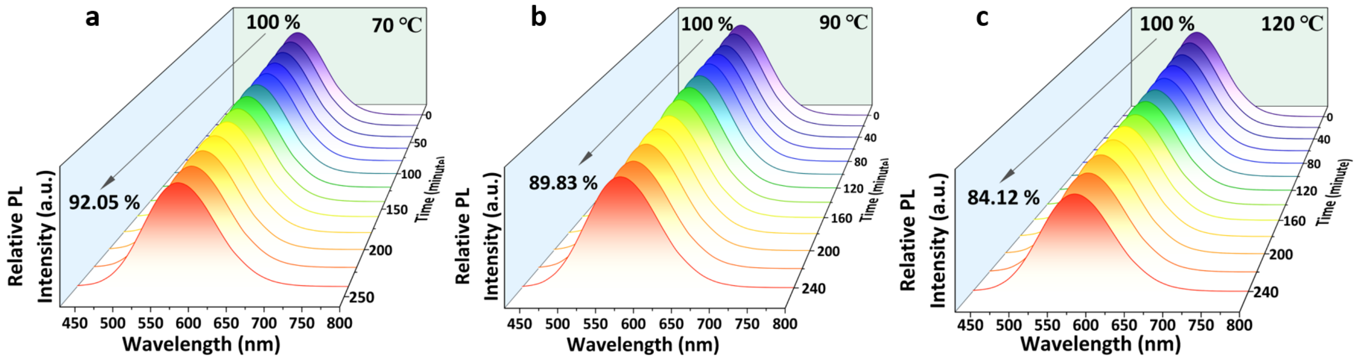

Figure S15. PL spectra of ODASnBr4 as a function of time in thermal stability test under (**a**) 70 °C, (**b**) 90 °C and (**c**) 120 °C.

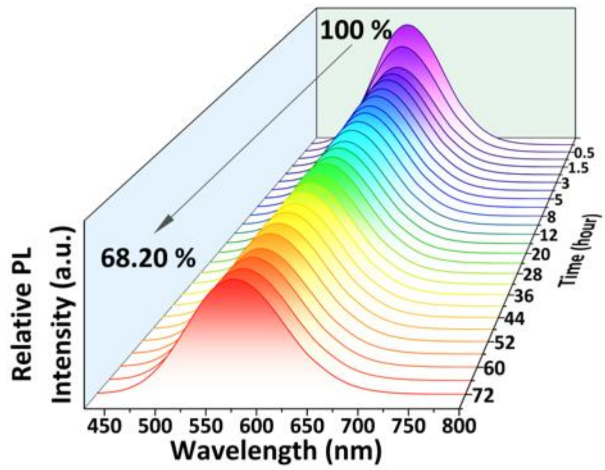

Figure S16. PL spectra of $ODASnBr_4$ as a function of time in the photostability test.

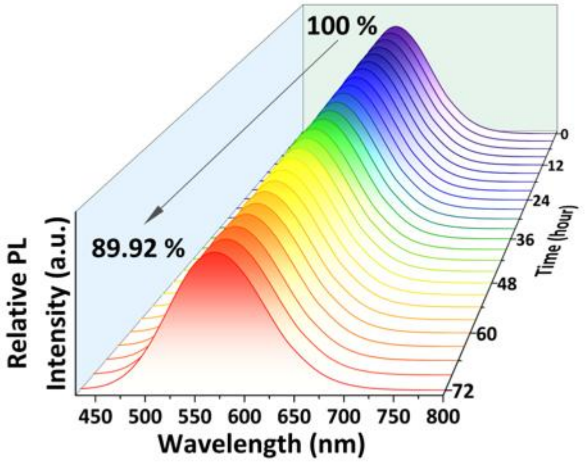

Figure S17. PL spectra of $ODASnBr_4$ as a function of time in the air stability test.

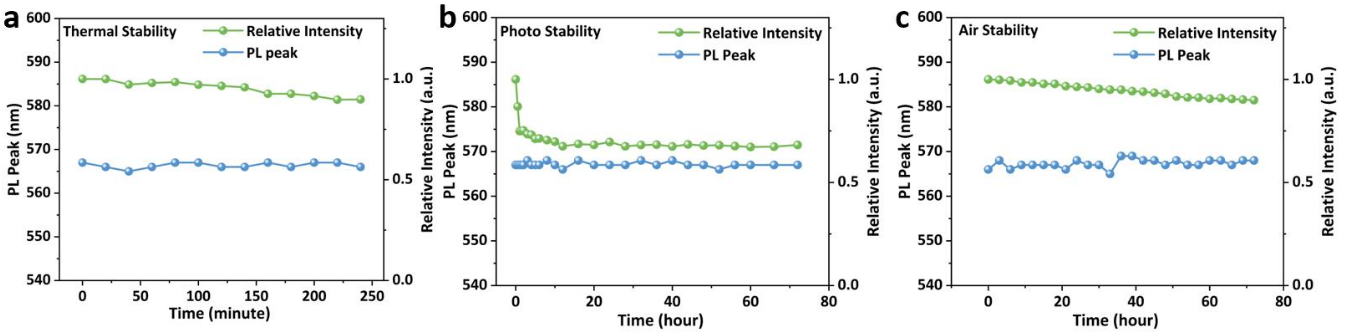

Figure S18. Relative intensity and PL peaks of $ODASnBr_4$ as a function of time in (**a**) thermal (90 ℃), (**b**) photo and (**c**) air stability tests.

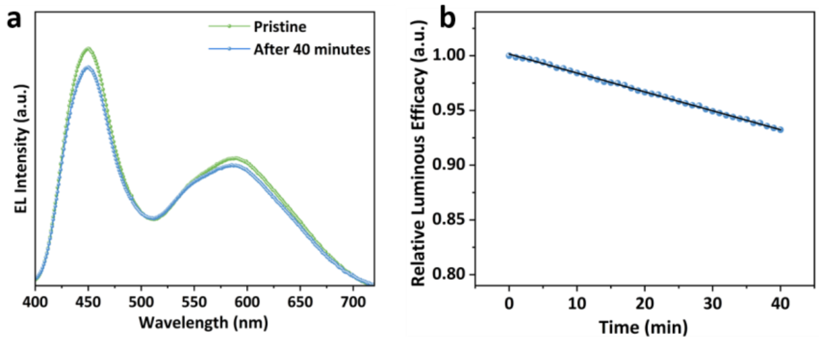

Figure S19. (**a**) EL spectra and (**b**) LE of the prepared WLED with a blending ratio of 2:1 before and after the stability test.

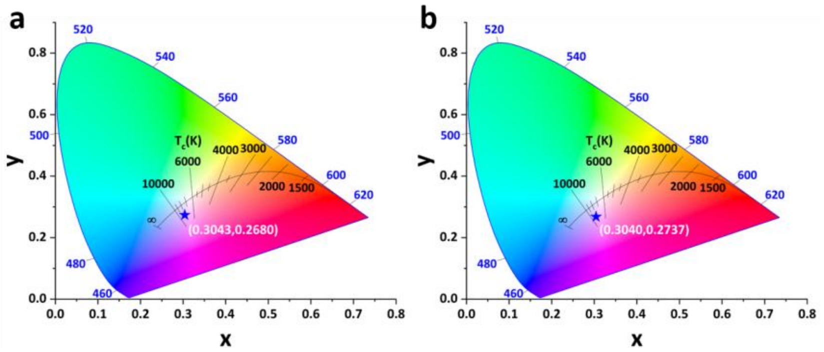

Figure S20. CIE chromaticity coordinates of the WLED (**a**) before and (**b**) after stability test.

Table S1. Fitting parameters in XPS spectra.

Materials	Peak Assignment	Binding Energy/eV	FWHM/eV
$ODASnI_4$	N 1s ($-NH_3^+$)	401.60	1.34
	N 1s ($-NH_2$)	399.92	1.01
	Sn $3d_{3/2}$ (Sn^{4+})	495.87	1.22
	Sn $3d_{3/2}$ (Sn^{2+})	494.63	1.09
	Sn $3d_{5/2}$ (Sn^{4+})	487.42	1.31
	Sn $3d_{5/2}$ (Sn^{2+})	486.19	1.11
	I $3d_{3/2}$ (I_2)	631.57	1.08
	I $3d_{5/2}$ (I^-)	630.25	1.25
	I $3d_{3/2}$ (I_2)	620.06	1.00
	I $3d_{5/2}$ (I^-)	618.76	1.25
$ODASnBr_4$	N 1s ($-NH_3^+$)	401.42	1.53
	N 1s ($-NH_2$)	399.43	1.22
	Sn $3d_{3/2}$ (Sn^{4+})	496.38	1.51
	Sn $3d_{3/2}$ (Sn^{2+})	495.31	1.65
	Sn $3d_{5/2}$ (Sn^{4+})	487.93	1.44
	Sn $3d_{5/2}$ (Sn^{2+})	486.88	1.63
	Br $3d_{3/2}$	68.80	1.31
	Br $3d_{5/2}$	67.77	1.16

Table S2. Percentages of the major elements calculated based on XPS spectra.

Materials	Element	Atomic/%
$ODASnI_4$	N	48.53
	Sn	9.85
	I	41.62
$ODASnBr_4$	N	48.40
	Sn	8.53
	Br	43.07

Table S3. Yield of $ODASnBr_4$ calculated by tin under different experimental conditions; "/" represents no production.

HBr/mL	H_3PO_2/mL	Sn^{2+} Precursor	Temperature/℃	Time/min	Yield/%
4	13.5	SnO	90	30	86.47
2	13.5	SnO	90	30	/
3	13.5	SnO	90	30	69.47
5	13.5	SnO	90	30	90.89
6	13.5	SnO	90	30	77.38
4	10.5	SnO	90	30	74.46
4	12	SnO	90	30	79.71
4	15	SnO	90	30	56.01
4	16.5	SnO	90	30	/
4	13.5	$SnOAc_2$[a]	90	30	58.91
4	13.5	$SnBr_2$	90	30	65.62
4	13.5	$SnOA_2$[b]	90	30	/
4	13.5	SnO	100	30	50.29
4	13.5	SnO	95	30	74.71
4	13.5	SnO	85	30	42.75
4	13.5	SnO	80	30	/
4	13.5	SnO	80	60	79.71

a: $SnOAc_2$ denotes stannous oxalate. b: $SnOA_2$ denotes stannous octoate

Table S4. Performance parameters of WLED prepared with $ODASnBr_4$ and BMA under a 2:1 blending ratio.

Driving Current/A	Luminous Flux /lm	Luminous Efficacy /lm W^{-1}	CCT/K	Ra	Color Coordinates
0.04	12	93.23	7754	87.1	(0.3104, 0.2644)
0.08	25.5	96.26	8052	88.9	(0.3068, 0.2675)
0.12	39	96.27	8226	89.9	(0.3045, 0.2699)
0.16	52.5	95.71	8222	90.7	(0.3033, 0.2734)
0.20	65.4	94.14	8226	91.1	(0.3023, 0.2761)
0.24	76.8	90.93	8344	91.3	(0.3005, 0.2782)
0.28	84.4	84.71	8714	91.6	(0.2971, 0.2780)
0.32	88	76.49	9568	91.8	(0.2917, 0.2735)
0.36	89.3	68.32	10430	91.9	(0.2875, 0.2693)
0.40	89.5	61	11500	91.9	(0.2833, 0.2647)
